# Taking a ‘Big Data’ approach to data quality in a citizen science project

**DOI:** 10.1007/s13280-015-0710-4

**Published:** 2015-10-27

**Authors:** Steve Kelling, Daniel Fink, Frank A. La Sorte, Alison Johnston, Nicholas E. Bruns, Wesley M. Hochachka

**Affiliations:** Cornell Lab of Ornithology, Cornell University, 158 Sapsucker Woods Rd., Ithaca, NY USA; British Trust for Ornithology, The Nunnery, Thetford, IP24 2PU UK

**Keywords:** Biodiversity monitoring, Citizen science, eBird, Data quality, Species distribution models

## Abstract

Data from well-designed experiments provide the strongest evidence of causation in biodiversity studies. However, for many species the collection of these data is not scalable to the spatial and temporal extents required to understand patterns at the population level. Only data collected from citizen science projects can gather sufficient quantities of data, but data collected from volunteers are inherently noisy and heterogeneous. Here we describe a ‘Big Data’ approach to improve the data quality in eBird, a global citizen science project that gathers bird observations. First, eBird’s data submission design ensures that all data meet high standards of completeness and accuracy. Second, we take a ‘sensor calibration’ approach to measure individual variation in eBird participant’s ability to detect and identify birds. Third, we use species distribution models to fill in data gaps. Finally, we provide examples of novel analyses exploring population-level patterns in bird distributions.

## Introduction

The conservation of species begins with an understanding of the patterns of distribution, abundance, and movements of individuals. These patterns are driven by an interacting series of climatic, geological, ecological, and anthropogenic processes operating simultaneously across a range of spatial and temporal scales (Bell 2012). Only by comparing these patterns across a range of spatial and temporal scales can we begin to identify the interacting role of these processes. For example, if a species–habitat association does not vary across a wide geographical area, we can gather data within a limited spatial extent and make inferences and predictions well outside the area of data collection. When species–habitat associations change across spatial or temporal scales, as they often do (Gaston and Spicer 2013), then making predictions requires a broader spatio-temporal perspective.

In general, to study and understand entire ecological systems, data must be collected at fine resolutions over broad spatial and temporal extents, particularly for wide-ranging species. However, the cost and availability of experts needed to collect sufficient quantities of ecological data do not scale readily across broad spatial or temporal extents. Citizen science projects have emerged as an efficient way to gather such data by engaging a large number of people and compiling their ecological observations, and the fastest growth in species’ distribution data comes from volunteers participating in citizen science projects (Pimm et al. 2014).

Nevertheless, data gathered by citizen science projects are often highly variable due to the opportunistic approach for data collection, which poses several challenges to its analysis and interpretation. First, engaging the large numbers of volunteers needed to collect data across broad extents requires data collection protocols that are straightforward and enjoyable, instead of complex and tedious (Bonney et al. 2009). The drawback to this approach is that it gives volunteers the choice of how, where, and when they make observations. In general, this results in more heterogeneous data that are less informative than data collected under more constrained data collection protocols (Hochachka et al. 2012). Second, open participation of a broad public will attract participants with varied skill levels at detecting and identifying organisms. Third, many citizen science projects fall into the category of surveillance monitoring, which is motivated by general data collection for many uses and lacks strong a priori hypotheses to shape the data collection protocol; this has, for example, led to criticism of surveillance monitoring for its lack of management-oriented hypotheses (Nichols and Williams 2006). The lack of well-defined hypotheses to define data collection protocols and individual variability in skill levels and data collection processes are major obstacles in accurately interpreting citizen science data that must be recognized and addressed during analysis.

Recent advances in Big Data, a broadly defined field encompassing the access, management, and computational processing of extremely large data sets to reveal associations, patterns, and trends (Manyika et al. 2011), are increasingly being integrated into ecological studies (Hampton et al. 2013). Big Data is not just about “a lot of data” but includes developing methods to handle the constant acquisition of new data, integrating disparate data from multiple sources, and most importantly addressing issues of data quality across the various sources of data (Lagoze 2014).

One citizen science project that is collecting large volumes of data across broad spatial and temporal extents is eBird (Sullivan et al. 2009), which uses Big Data techniques to curate, access, and analyze data. The goal of collecting information about birds’ distributions across huge regions and throughout the year requires eBird to engage a large number of regular participants; this is only possible when participants are not highly constrained in how they make their observations (Wood et al. 2011). However, these same protocols impose a cost during the data analysis because it is easier to analyze data that *conform* to more standardized protocols that remove potential sources of variation in counts of birds by constraining aspects of the observation process, for example, locations, times of day, and durations of observation. The analysis challenge is the need to identify and model important aspects of the observation process that were not controlled during data collection. Once sources of variation in the observation process can be modeled, they can be predicted, which provides the avenue for post-collection analytical data quality control.

In this paper, we address the data quality challenges inherent in surveillance monitoring projects such as eBird. First, we describe how we improve data quality during data submission. Second, we show how we can model variability among individual participants. Third, we describe how we use species distribution models to fill in data gaps while also modeling the data collection process in order to control for biases in where, when, and how eBird data are collected.

## Materials and methods

### eBird

Data for this study came from eBird, which engages volunteers via the Internet and mobile apps to collect bird observations (Sullivan et al. 2014). Presently, more than 250 000 participants have submitted more than 17 million checklists that include 260 million bird observations from all countries globally. While the majority of observations are from the Western Hemisphere, eBird has recorded 97 % of the world’s known bird species. All eBird data are stored within a well-curated, and accessible, data repository (Kelling 2011).

### Observation process information

Data collection for eBird is based on how birders typically go into the field to observe birds. Typically, a birder will go to a specific location (i.e., a preserve, park, or watch site) and either walk a transect to observe birds, or stand at one location and observe the birds that pass by. Often, birders keep records, in the form of checklists, which include the date, start time, and location they made their observations as well as a list, which often includes counts of individuals, of the species they observed. Each eBird observation contains seven data collection covariates that provide information on how eBird participants collected their observations. These covariates identify the observer, the location the observations were made, the duration spent searching for birds, the distance traveled during a search (which could be zero if the observer stood at one location), the number of individuals of each species observed, the number of people in the search party, and whether they were submitting a complete checklist of all the birds that they observed or only a partial list of the species they observed (Sullivan et al. 2009). These covariates are used to account for variation in detectability associated with search effort.

### Ecological process information

To account for the effect of habitat distribution on patterns of species observations, we include covariates that describe topography and land cover at search locations. The latitude and longitude is stored in the database for every search location. We linked these locations with elevation calculated from the ASTER instrument onboard NASA’s *Terra* Satellite (Tachikawa et al. 2011), and land cover based on the MODIS global land cover product (MCD12Q1) (Friedl et al. 2010). The University of Maryland classification scheme (Hansen et al. 2000) is used for habitat categories. For eBird species distribution modeling, each 500 m × 500 m MODIS pixel is classified into one of 14 land cover classes, which are summarized as proportions within a 3 km × 3 km (900 ha) set of pixels centered at each eBird observation location.

### Data organization

Data used here come from the eBird Reference Data set (Sullivan et al. 2014), available on the eBird website.^1^ This data set includes complete checklists (i.e., observers indicate that they reported all the birds they observed) of counts of individual birds by species including zeros implied for unreported species, information about the observation process, and information about the local environment where each search took place. Details on the structure of the eBird data set and methodology used to link eBird observations with environmental variables are available, see (Munson et al. 2009).

### Improving data quality during data submission

The eBird project has developed methods to ensure that data conform to basic quality standards during data submission. All eBird records are gathered as checklists of the presence or (in the vast majority of cases) counts of birds of all reported species, and all information on the observation process (Sullivan et al. 2009). Prior to a checklist being accepted in the eBird database, the observer must enter all data on the observation process during submission. Additionally, all species reported on the checklist are passed through data quality filters that rely on historic eBird data and expert opinion, and any unusual records are flagged for further review (Kelling et al. 2011). Notification is provided immediately to the submitter to help establish whether information was entered correctly. If the submitter believes their flagged record to be accurate, it is sent to one of more than 950 regional experts for review. In any given year, approximately 5 % of all observations submitted are flagged for review (i.e., more than 3.5 million observations were reviewed in 2014) and approximately half of those records flagged are marked as invalid in the eBird database. This combination of artificial intelligence (the data quality filters) and human intelligence (expert reviewers) creates an active learning feedback loop between humans and computers that dramatically improves the data quality prior to the data being integrated into the eBird database (Kelling et al. 2013).

### Improving data quality after submission

After data entry into the eBird database and any data reviews have been completed, the next challenge is to account for sources of data variation that arise from the non-standardized aspects of data collection. We discuss two categories that are likely of greatest relevance: (1) variability in observer skill and (2) the non-uniform distribution of search effort across space and time.

### Measuring observer variability

An important part of extracting biological signal from eBird’s data is using analyses that control for the large variation between eBird participants in their ability to detect and correctly identify bird species. To estimate variability in observer skill, we developed individual data submission profiles to rank eBird participants based on their species accumulation rates—the rate at which new species are added to a checklist with increasing time spent during an observation period.

We start with the presumption that any given geographic region contains a finite number of species, and the number of species detected and identified will approach an asymptote as the length of the observation period increases (Fisher et al. 1943). For our purposes, we have used as our geographic regions the North American Bird Conservation Regions (BCRs); these regions delineate logical biogeographic units and have been widely adopted for bird research and conservation efforts (Sauer et al. 2003). Data submission profiles for each observer within each BCR will be affected by three categories of variables: (1) the total number of species present, (2) characteristics of the birds themselves and the observation process for an average observer that affect the probability of species detection, and (3) observer-specific deviations from the average ability to detect and identify species. We selected relevant covariates to account for sources of variation in categories 1 (e.g., time of year and habitat) and 2 (e.g., time of day and effort spent birding) to control for known sources of variation that will affect all observers and leave the remaining observer-specific variation from which to create each observer’s data submission profile (category 3). We then modeled the number of species on a checklist, so that we were able to describe how the number of species recorded changed with the length of the observation period—a relationship typically termed a species accumulation curve. The resulting model allowed us to create species accumulation profiles specific to each observer, from which an index of observer expertise could be calculated. In this paper, the index of observer expertise is the number of species expected for a checklist from a 1-h observation period.

### Using species distribution models to address uneven sampling

While the quantity of data in eBird makes it one of the largest biodiversity data sets, the distribution of search effort across space and time is irregular, making it difficult to accurately infer patterns of occurrence or abundance from the raw data, particularly in areas or during specific times of year where observations are sparse. Even in regions that have higher densities of data, variation in search effort among checklists means that records of species on checklists have the biological information about occurrence and abundance confounded with variation in the likelihood observers detected individual birds that were actually present. To overcome these challenges in raw eBird data, we use species distribution models (SDMs) (Franklin 2009) to simultaneously “fill-in” geographical gaps between locations of data collection and “standardize” search effort to correct for variation in detection rates from checklist to checklist.

Our models are based on the fact that each species has an environmental niche and identify associations between local environmental features and the probability of presence or abundance of a species. We obtain data describing local environments at all of the locations for which we have checklists of bird observations, and build SDMs to identify relationships between habitat features and presence or abundance of a species at checklist locations. These same SDMs also describe how either probability of reporting or abundance varies with variation in the observation process (e.g., duration of observation period). We are then able to predict the probability of occurrence or abundance of a species at any location that we want, based on the habitat at these locations and a standardized set of observation conditions (duration of observation period, time of day, distance traveled, number of observers). In future iterations of the SDM, we plan to incorporate variation in observer skill, as preliminary results indicate that including individual species accumulation profiles improves the accuracy of SDMs. In essence, rather than collecting bird data in a systematic fashion from a massive number of locations across a region, we use an SDM to describe how the data should have looked if we had conducted such a massive and systematic survey.

eBird collects data from broad regions and throughout the year, and we want to take advantage of these data to describe complex annual patterns of species’ occurrence and abundance. In creating a single model that can describe variation in occurrence or abundance, and variation through time, we needed to overcome the problem of statistical non-stationarity (Finley 2011): variation among regions and through time in a species’ habitat preferences. To solve this problem, we developed a two-stage SDM called the spatio-temporal exploratory model (STEM) (Fink et al. 2010, 2014). Briefly, the two stages are the building of a very large number of ‘local’ SDMs with each local model built on data from a smaller and arbitrary region and time period, and then combining these local models into a single ‘ensemble’ model that covers the entire large region and calendar year. When regions and time periods for the local models are appropriately chosen, statistical non-stationarity is not an issue within each local model’s data. Because the regions and time periods overlap across the local models, the set of local models can be stitched together into a seamless ensemble model that describes distribution or abundance at any location and at any time of year.

In our use of STEM models to describe distributions of birds across the North American continent, we do not make predictions at all possible locations and dates. Instead, we calculate probabilities of occurrence at weekly intervals throughout the year and at a randomly chosen location within a regular 3 km × 3 km grid across all of North America. Because STEM models describe how detection of birds changes with a set of predictor variables, we need to make these predictions for one arbitrary set of observation conditions in order to extrapolate across locations and dates. Currently, these conditions are for a count to have been conducted from 7 to 8 a.m. by a single observer who traveled 1 km in the making of their count.

## Results

### Measuring observer variability

**Fig. 1:**
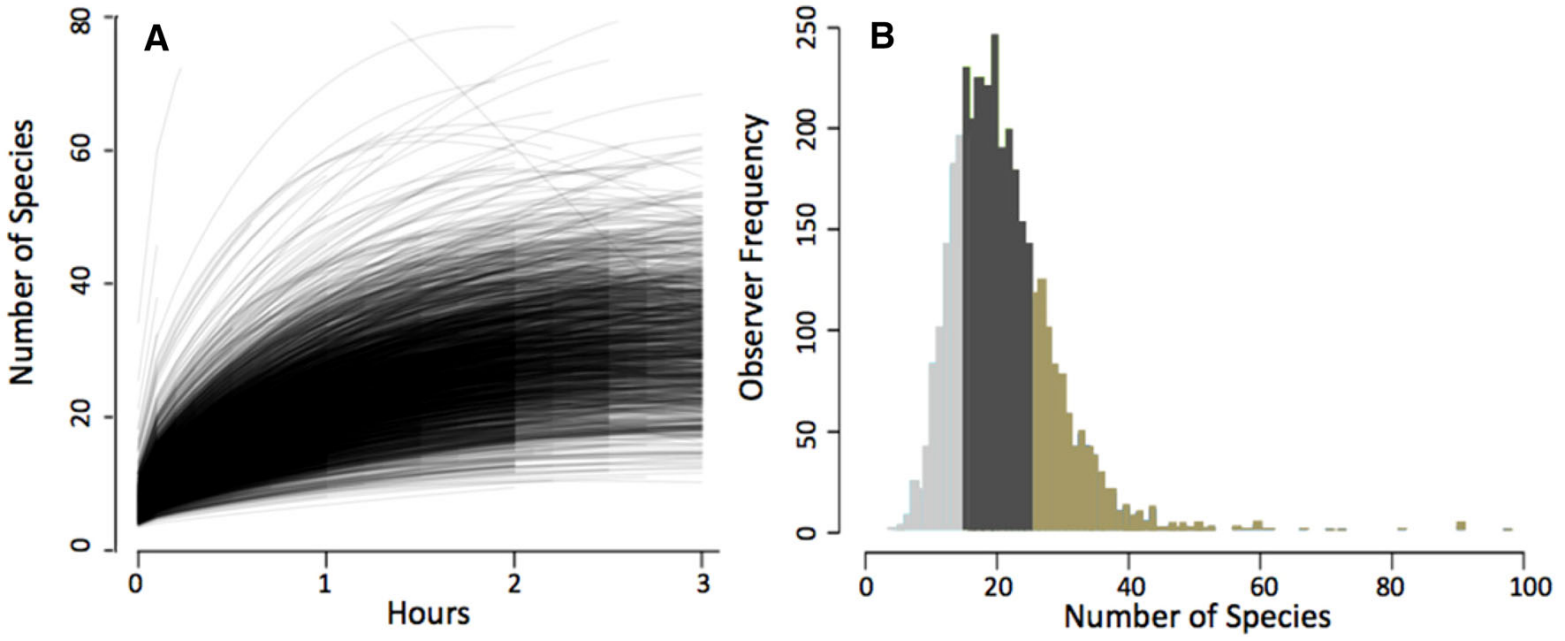
**a** Variation in species accumulation curves for all 3660 observers in Bird Conservation Region 30. As the duration an observer spends collecting data increases, the number of species observed increases and the rate of species accumulation decreases. **b** The number of species observed in 1 h for all observers in BCR 30. The *light gray* region represents the lower quartile of novice observers, and the *brown* region the upper quartile of expert observers

**Fig. 2:**
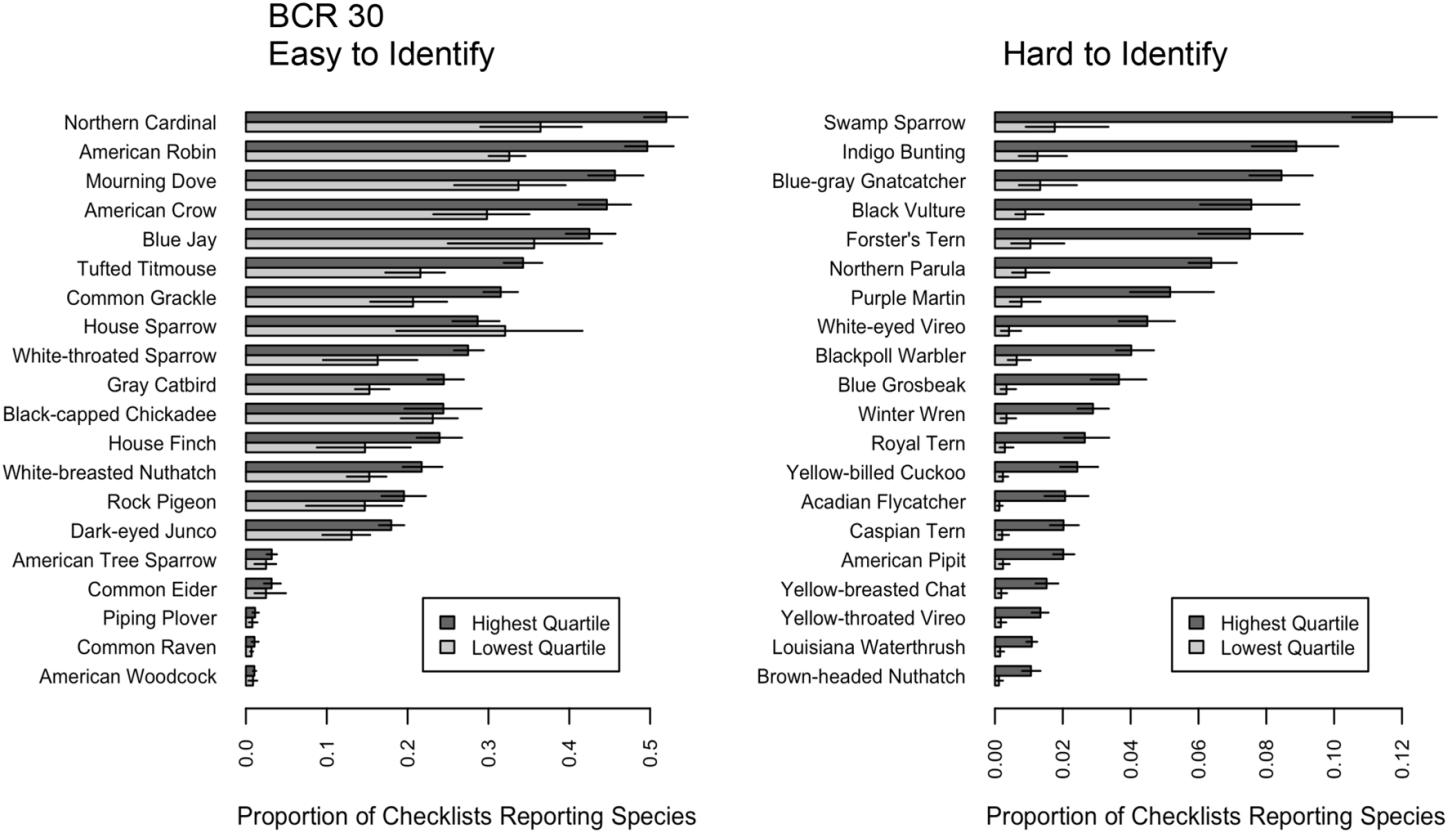
Comparisons between detection rates of the highest and lowest quartiles of eBird observers in BCR 30 (see Fig. 1b). Detection rates are indexed as the proportion of checklists on which each species is reported (*error bars* are 95 % bootstrap confidence intervals), and as such are only useful for making comparison between the two groups of observers within a species

To illustrate the results of our estimates of observer variability, we present results from observations made in Bird Conservation Region 30, the New England and Mid-Atlantic Coast of the United States. A total of 3660 participants submitted 312 987 checklists in BCR 30. Typical of observers from other regions, we found high among-observer variation in their species accumulation rates (i.e., profiles) (Fig. 1a). From these curves, we derive individual species accumulation profiles, the expected number of species observed in an hour, in order to illustrate the variation in observer expertise (Fig. 1b). We examined whether there were consistent differences in the types of observations made by observers in the highest and lowest quartiles based on the individual profiles (Fig. 2). Barplots were generated for the 20 species where the detection rates are proportionally most similar between the two quartiles (Fig. 2 left) and the 20 species for which detection rates are proportionally most different. The 20 species for which the two groups have proportionally most similar detection rates are generally species that are fairly easy to identify by sight. The 20 species that the two groups have proportionally most different detection rates are generally species that are difficult to identify, easier to identify by sound, or often be seen as a high-flying silhouette without many distinguishing features. Additionally, members of the higher quartile submitted more checklists per observer (mean number of checklists was 126 per observer for highest quartile and 36 checklists per observer for lowest quartile). These results indicate that a significant subset of eBird participants not only has greater expertise in detecting and identifying birds but also submits the majority of data to eBird.

### Species distribution estimates

**Fig. 3:**
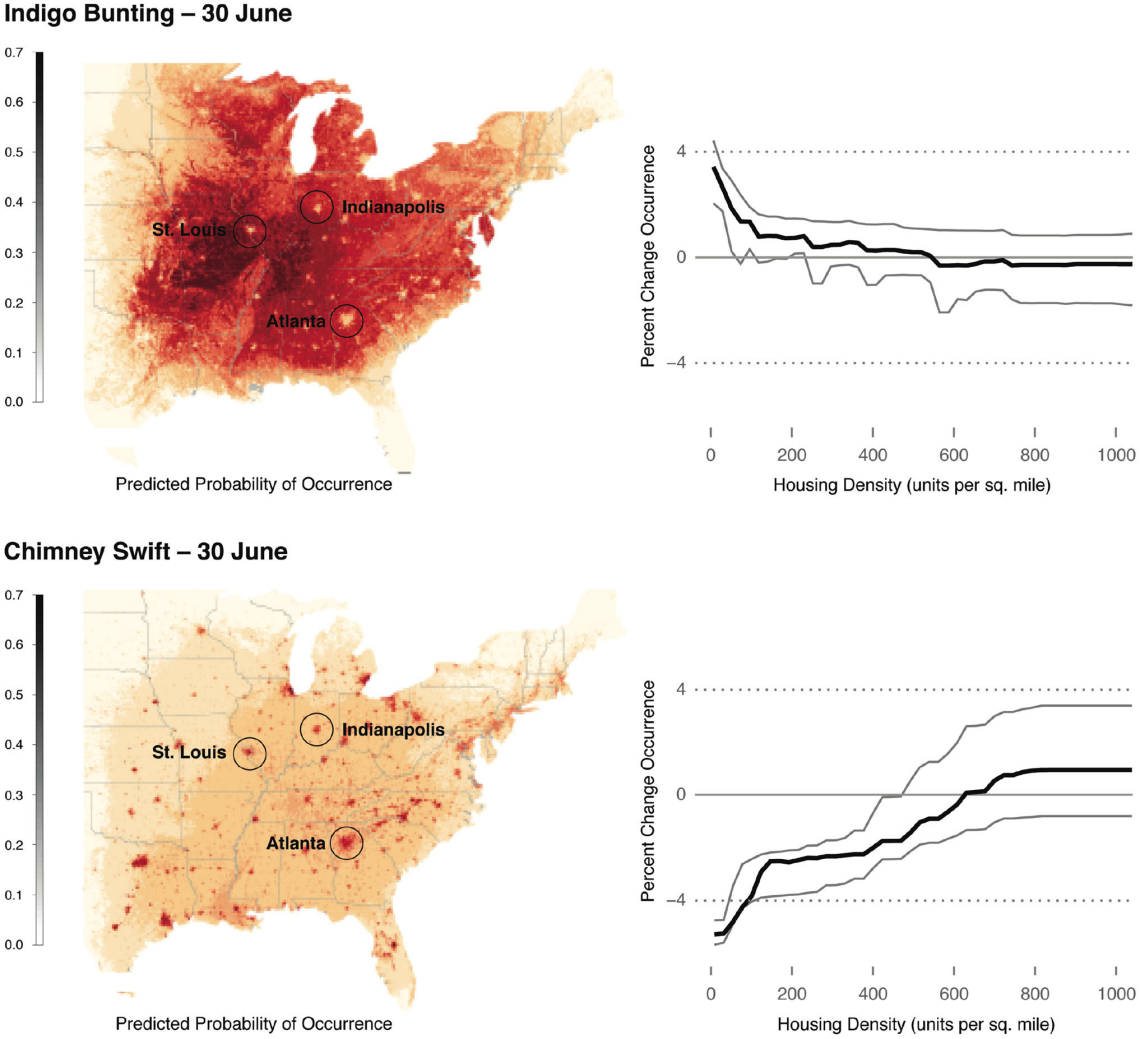
Predicted probability of occurrence and partial dependence on housing density for Indigo Bunting (*top*) and Chimney Swift (*bottom*) for June 30. Although both species have a widespread distribution across eastern U.S., the fine-scale differences around major urban centers are striking (e.g., note the three *highlighted* urban centers, Indianapolis, St. Louis, and Atlanta). The Indigo Bunting requires natural forest and shrub habitats for breeding and has relatively low occurrence rates in urban centers, while the Chimney Swift nests in chimneys have relatively high occurrence rates in urban centers

The STEM describes bird species’ distributions across any region at any time of the year, while controlling for important sources of observation variability such as search effort. Here we present illustrations of the model’s ability to describe ecological patterns and the types of results that can be extracted in order to gain ecological insights. Figure 3 shows the breeding occurrence and habitat preferences of Indigo Bunting (*Passerina cyanea*) and Chimney Swift (*Chaetura pelagica*), two species with similar breeding ranges. Both prior knowledge of the patterns of these species occurrences and more quantitative measures of predictive performance (i.e., Area-under-the-Curve estimates (Fielding and Bell 1997)) provide evidence for the accuracy of these estimates. Finally, the contrasting partial effects (Friedman 2001, Hastie et al. 2009) of Housing Density between the two species in the same area at the same time of the year are plotted in Fig. 3 (right). After accounting for effects of search effort—the models describe the avoidance of areas of high human density by Indigo Buntings and the contrastingly high occurrence rates of Chimney Swift in urban centers. We emphasize that the distribution maps are based on habitat associations identified using STEM, and not simple interpolations.

**Fig. 4:**
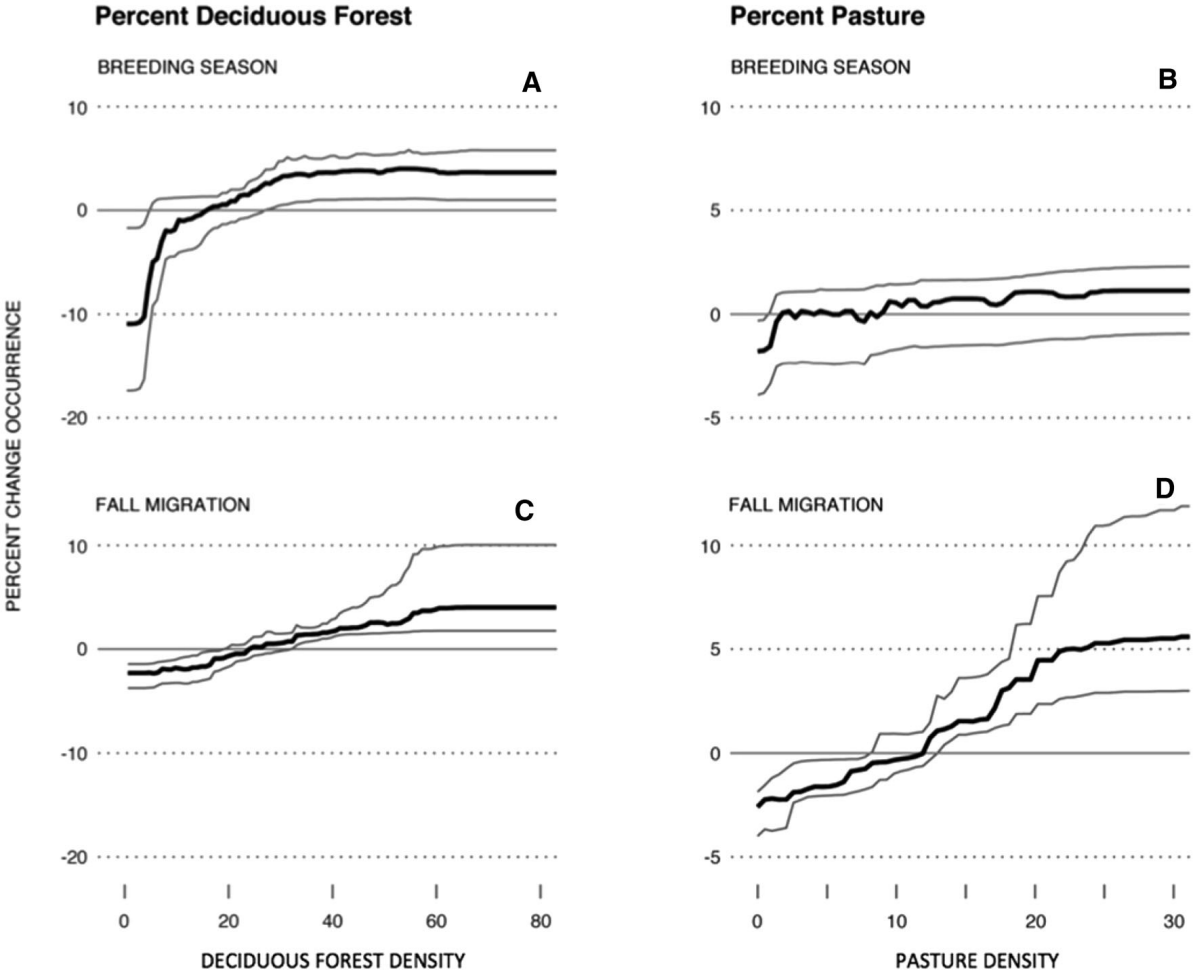
Seasonal differences in associations of Indigo Buntings with individual habitat features (means with 95 % confidence intervals) while controlling for the effects of additional predictors of distribution during the breeding season (June 5–July 31) and during fall migration (September 1–October 15). All associations with habitat (as estimates by percentages of a habitat type within a 225 ha region around a location) were estimated using partial dependence functions. The partial effect of deciduous forest is for stronger association of Indigo Buntings with deciduous forest during the breeding season (**a**, **b**) than during the fall (**c**). The essentially *horizontal line* in (**c**) indicates no significant partial effect of pasture in spring, but a strong preference for areas with high amounts of pasture during the fall migration (**d**)

STEM models allow us to control for both observer variability in effort (e.g., the duration of birding) and location (i.e., eBird data are disproportionately submitted near roads and populated areas). The result is that STEM models improve the quality of the interpretations we can make from eBird data. For example, our use of STEM to create a single year-round model of distributions has been valuable for discovering seasonal species’ ecological niches, and the generality of these changes across entire distributions of species. Figure 4 demonstrates our ability to detect and describe population-level seasonal changes in habitat associations. The partial effect of the percent of deciduous forest has a strong positive effect on Indigo Bunting occurrence rates during the breeding season and a slightly weaker positive effect during autumn migration. In contrast to the breeding season, areas with a greater proportion of pasture appear to be preferred during autumn. Indigo Buntings often nest on edges of hardwoods where insects comprise much of their diet; during the winter, they occur in more open agricultural areas where their diet is composed primarily of seeds (Payne 2006). Our models suggest that the Indigo Bunting begins a shift to winter habitat associations soon after breeding and prefers more open habitat during autumn migration.

**Fig. 5:**
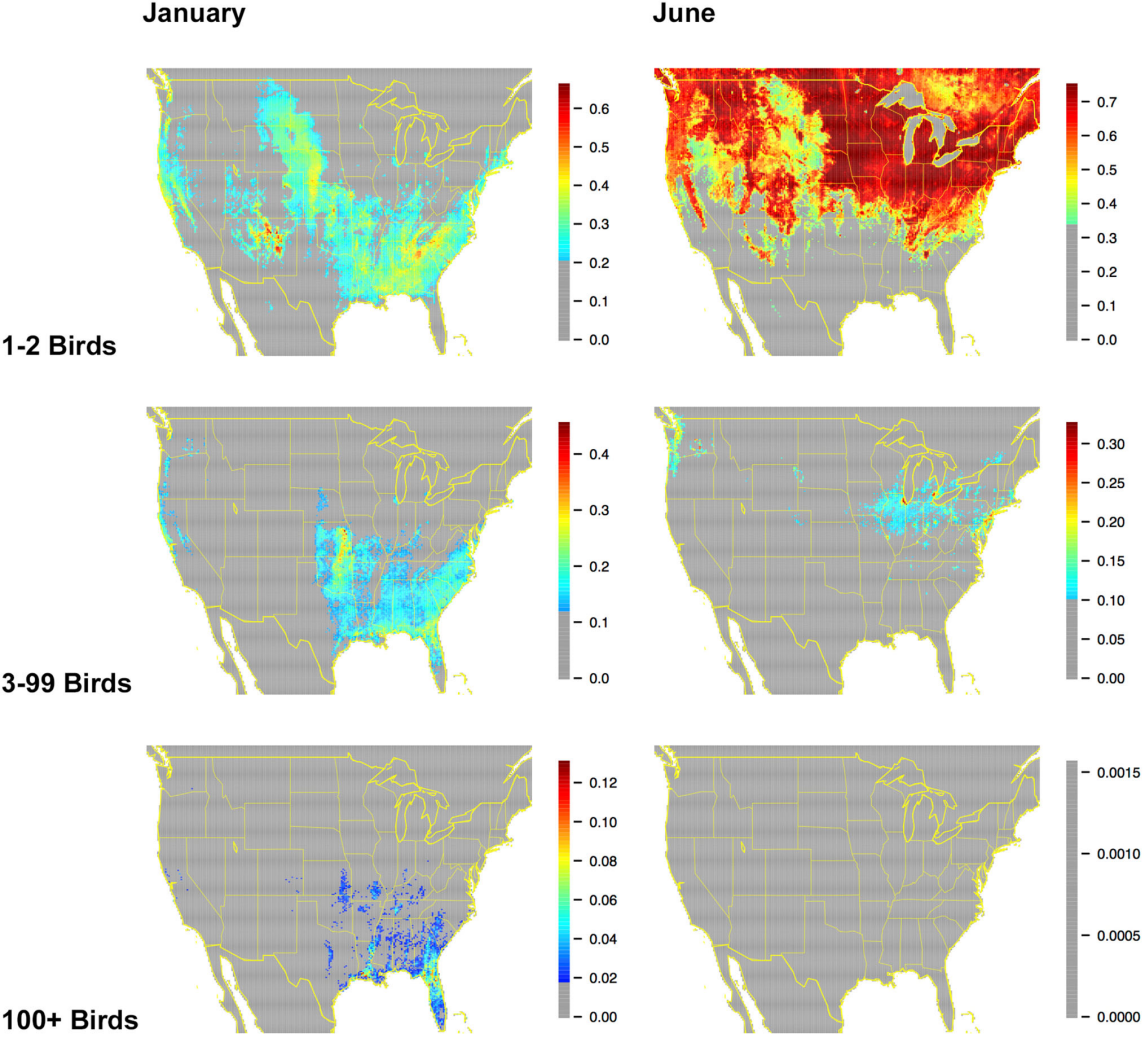
American Robin abundance for January and June, as described by a multinomial logistic regression. This procedure treats abundance across a small set of ranges that are appropriately chosen for the species at hand (here: 0 birds, 1–2 birds, 3–99 birds, and 100+ birds) and creates a set of models that describe the probability that a checklist will contain a count in each of the pre-chosen abundance classes. All estimated probabilities falling below thresholds were ‘*grayed out*’ in the figures

While most of our analyses to date have focused on modeling probabilities of occurrence, we are currently exploring the use of the same STEM framework to model relative abundance. Figure 5 shows an example of this work, using a form of abundance modeling (multivariate logistic regression) in which abundances are described using a small set of abundance classes, with the probability of observation of numbers of birds within each class being described by the model. As in the results presented for the occurrence estimates, these distribution estimates use STEM to standardize the effects of search effort as the probabilities that a typical eBird participant would count enough individual American Robins (*Turdus migratorius*) for each abundance class on a search conducted from 7 to 8 a.m. while traveling 1 km at the given location and time of year. The STEM analysis shows that in both January and June small numbers of individual American Robins are predicted to occur over larger regions than were large numbers of individual birds. The largest counts of 100+ birds were essentially only in winter, a time of year when American Robins are known to form large flocks.

## Discussion

Given the large quantities of data that can be collected by citizen science projects, Big Data methods for data management, quality assurance, and data analysis are invaluable to the process of using citizen science for research. In this paper, we have presented descriptions of Big Data methods used for one citizen science project, eBird, to illustrate this claim. First, eBird’s data management strategy ensures that all data submitted meet high standards of completeness and accuracy during data input. Second, we have taken a ‘sensor calibration’ approach to measure individual variation in eBird participant’s ability to detect and identify birds. Third, we have developed species distribution models that accurately describe patterns of distribution of birds across broad spatial and temporal scales at very high resolution.

### Improving data quality during data submission

Much effort is made to ensure high standards of record completeness and accuracy during data submission to eBird, and more than 75 % of eBird checklists include all effort information. Next, we combine both expert opinion and artificial intelligence into a human/computer network to create data filters that screen data submissions at the time they are submitted. While we have not presented any information in the Results section illustrating the effectiveness of our data quality processes at data input, we note that they are effective enough to take an off-the-shelf data product and use it directly for the analyses presented in this paper without the need for additional data screening. For the data analysts associated with the eBird project, this is a very novel and welcome change from the standard workflow of analyzing data that involve a major initial component of post hoc quality control checking and manual fixing of the data. These data are openly downloadable as eBird Reference Dataset (http://ebird.org/ebird/data/download).

### Measuring observer variation

By examining data coming from observers with a range of skills, we can characterize how skill levels differ among observers. Our preliminary results, from one Bird Conservation Region in the United States, indicate that those observers who submit the most observations to eBird not only submit more checklists, but also report birds that are harder to identify. Further analysis indicates that this pattern is consistent in other regions. This has allowed us to conclude that the trade-off between data quality and data quantity in eBird is less than previously thought. Finally, preliminary evidence indicates that individual species accumulation profiles could be used in the future to help ‘manage’ the issue of differential ability when estimating detailed spatio-temporal species occurrence or abundance estimates.

While the calibration of sensors is a standard procedure for large networks of autonomous sensors (Rachlin et al. 2011), we have illustrated how the data already collected by participants in eBird contain the data needed to calibrate our human ‘sensors.’ This Big Data approach of retrospectively improving data quality by building calibration models differs from alternative approaches used in citizen science projects such as extensive training and testing of participants (Kendall et al. 1996), or having multiple participants undertake the same task (Siddharthan et al. 2015). We suggest that there is a wider potential for using post hoc calibration in analysis of data from citizen science projects.

### Species distribution models

The Spatial Temporal Ensemble Model (STEM) produces analytically tractable summaries of surveillance monitoring data. We feel that, from a Big Data perspective, the most important aspect of our analyses of eBird data is the methods developed and employed to identify patterns in the data without intensive and continuous attention from data analysts. These methods are in contrast with the standard statistical analytical methods taught to ecologists, which require considerable ecological insights prior to analyses (Hochachka et al. 2007). It would have otherwise been logistically impossible for us to conduct the huge number of analyses, including the SDMs for hundreds of species of birds, needed for uses such as providing the results for the United States of America State of the Birds reports (North American Bird Conservation Initiative 2011, 2013).

The species distribution models we have created are by their nature exploratory analysis methods (Kelling et al. 2009). From the biological perspective, this means that Big Data methods can be used to generate ecological hypotheses regarding associations of birds with their environments that can subsequently be tested (Strube et al. 2008). For example, we have developed a novel top-down investigative strategy to document patterns and test hypotheses from eBird and statistical products from STEM (La Sorte et al. 2013). With this strategy, scientific questions are first addressed at broad geographic and taxonomic extents with temporal and, in some cases, spatial details preserved. Here, evidence for relationships is ascertained with the knowledge that patterns at these scales may contain sources of variation that can operate in different fashions across scales. Thus, when consistent broad-scale patterns, even if weak, are identified, they are likely worth investigating. For example, seasonal variation in how migrants associate with prevailing winds (La Sorte et al. 2014a) and ecological productivity (La Sorte et al. 2014b) can be detected at continental extents. The next step in the strategy is to scale-down questions geographically and taxonomically to study finer-scale sources of variation, a process guided by the inferences generated within the broader perspective. For example, environmental conditions within specific geographical regions can be explored across taxonomic guilds, providing insights into how associations documented at broader scales are structured across migrants at regional scales (La Sorte et al. 2015). In some cases, this method may explain more variation than observed at the broadest scales, and sometimes it will simply partition the broad-scale variation into finer-scale components. In either case, there is the opportunity to test interesting hypotheses to reveal underlying mechanisms.

From a conservation perspective, STEM models can be used to direct land management decisions. Detailed knowledge of spatial and temporal variation in bird occurrence over broad spatial extents can only be obtained through surveillance monitoring projects like eBird. However, because of the variation in observer effort in raw eBird data, STEM is necessary to fill in the geographical gaps and create a uniform predictive surface at high spatial (1.5 km grid) and temporal (weekly) resolutions that can provide land managers with specific information on the timing of occurrence and distribution of bird populations. For example, the Nature Conservancy of California (TNC) needed to identify the best habitat available for waterbirds during migration. In order to reduce costs and increase available bird habitat, TNC devised a market-based approach to pay rice farmers in Central California to flood their fields for an extra 4–6 weeks during bird migration. To optimize this approach, eBird STEM models allowed TNC to identify when and which fields had the highest projected species abundance so as to prioritize which fields to lease for flooding during migration. Overall, more than 10 000 hectares of high-quality shorebird habitat were leased during the time when shorebird numbers were highest with resulting costs a small percentage of purchase or setting up a conservation easement.

## Footnotes

1 http://ebird.org/ebird/data/download.
